# Extreme Energy Density Confined Inside a Transparent Crystal: Status and Perspectives of Solid-Plasma-Solid Transformations

**DOI:** 10.3390/nano8070555

**Published:** 2018-07-21

**Authors:** Eugene G. Gamaly, Saulius Juodkazis, Andrei V. Rode

**Affiliations:** 1 Laser Physics Centre, Research School of Physics and Engineering, The Australian National University, Canberra ACT 2601, Australia; 2 Centre for Micro-Photonics, Swinburne University of Technology, Hawthorn VIC 3122, Australia; sjuodkazis@swin.edu.au

**Keywords:** light-matter interaction, ultra-short laser pulses, high-pressure/density conditions, phase transitions

## Abstract

It was demonstrated during the past decade that an ultra-short intense laser pulse tightly-focused deep inside a transparent dielectric generates an energy density in excess of several MJ/cm${}^{3}$. Such an energy concentration with extremely high heating and fast quenching rates leads to unusual solid-plasma-solid transformation paths, overcoming kinetic barriers to the formation of previously unknown high-pressure material phases, which are preserved in the surrounding pristine crystal. These results were obtained with a pulse of a Gaussian shape in space and in time. Recently, it has been shown that the Bessel-shaped pulse could transform a much larger amount of material and allegedly create even higher energy density than what was achieved with the Gaussian beam (GB) pulses. Here, we present a succinct review of previous results and discuss the possible routes for achieving higher energy density employing the Bessel beam (BB) pulses and take advantage of their unique properties.

## 1. Microexplosion Studies with Gauss-Shaped Beam

The studies of confined microexplosions during the last decade revealed the major features of this complicated phenomenon where the processes of electro-magnetic field/dielectric interaction, plasma formation and high-pressure hydrodynamics are intertwined. The concise description of these processes is as follows. The tight focusing of the laser beam deep inside a transparent crystal allows achieving the absorbed energy density in excess of the strength of any material in a sub-micron volume surrounded by the pristine solid. After energy transfer from hot electrons to ions, the expanding strong shock wave accompanied by the rarefaction wave starts propagating outside of this volume. After the shock decelerating and stopping, the void, surrounded by a shell of compressed and pressure modified material converted to the novel phases, is formed. All transformed material remains confined inside the bulk of undamaged material ready for further studies. These studies employed the short intense laser beam with the Gaussian spatial and temporal intensity profile [1,2,3,4].

The short intense laser pulse with the Gaussian spatial and temporal intensity profiles tightly focussed inside a transparent crystal generates an energy density of several MJ/cm${}^{3}$. The pressure produced is in excess of a few TPa, which is higher than the strength of any existing material (diamond has the highest Young’s modulus of 1 TPa = 1 MJ/cm${}^{3}$). The laser pulse, 150 fs, 100–200 nJ, 800 nm, tightly-focussed inside sapphire with a microscope lens (NA=1.4) creates the solid density plasma at the temperature of a few tens of electron Volts (∼$5\times 10^{5}$ K) with the record-high heating rate of $10^{18}$ K/s [1,2]. It was found that the novel (previously unobserved) high-pressure phases of aluminium and silicon were formed [3,4] following the ultrashort laser-induced confined microexplosion. Pressure/temperature conditions created in the microexplosion are similar to those in hot cores of stars and planets (“primeval soup” or warm dense matter). The material converted to high pressure/temperature solid density plasma is then transformed into the novel solid phase during the ultra-fast cooling and re-structuring. The major difference from the core-star conditions is the record-fast cooling (∼$10^{16}$ K/s) from plasma state to solid state. In the previous experiments, the study of the pressure-affected materials was produced postmortem, well after the end of the pulse when transformed material was cooled down to the ambient conditions. The structure of laser-transformed material was determined by the synchrotron X-ray diffraction [3] and with the electron diffraction [4] in transmission electron microscopy (TEM) [4].

### 1.1. Novelty of the Phase Transformation Path during and after Confined Microexplosion

The solid transforms to a solid-density plasma state ($T_{e}∼50$ eV) during a pulse time shorter than all energy relaxation times. A strong shock wave (SW) starts propagating from the energy deposition region several picoseconds after the pulse due to energy transfer from electrons to the ions. The shock wave decelerates and converts into a sound wave in the surrounding cold pristine crystal. The phenomenon is similar, but not identical to an underground nuclear explosion: the massless energy carriers (photons) deliver the energy inside a transparent crystal without changing the atomic and mass content of a material. All laser-affected material is expelled from the energy deposition area by the combined action of shock and rarefaction waves, forming a void surrounded by the shell of material compressed against the surrounding cold pristine crystal. The material returns from the high-pressure plasma state (high entropy, chaotic) to the ambient conditions at room temperature/pressure, however attaining a phase state different from the initial solid state. In all known methods of high pressure phase formation, the initial crystalline structure is re-structured, i.e., the atoms are moved from the initial arrangement to the new positions under the action of high pressure. During the transformation path under confined microexplosion, the initial state of a crystal is completely destroyed and forgotten. The irradiated material is converted into a chaotic mixture of ions and electrons at high temperature. Therefore relaxation to the ambient conditions occurs along the unknown paths going through the metastable intermediate equilibrium potential minima. The theoretical (computational, modified DFT-studies) during the last decade searched for the possible paths of material transformations under high pressure from the initially chaotic (stochastic) state [5]. These studies uncovered many physically allowed paths for the formation of multiple novel phases (including incommensurable phases) from the initially chaotic state. The confined microexplosion method now is the only practically realised way for the formation of novel material phases from the plasma state, preserving the transformed material confined inside the pristine crystal for further structural studies.

### 1.2. Limitations of the Confined Microexplosion Method with the GB

There are limitations to the energy density and amount of laser-affected material in confined microexplosion generated by the tightly-focused Gauss beam. The main limitation is imposed by diffraction: the radius of the diffraction-limited focal spot is [2,6]: $r_{Airy}=0.61\lambda /NA$; which defines the central Airy disk at the focus. For λ=800 nm and NA=1.4, one gets $r_{foc}=0.35\;\mu$m and a focal area of 0.38μm${}^{2}$. The absorption length in dense plasma equals ∼30 nm, giving the energy deposition volume ∼$10^{-14}$ cm${}^{-3}$. With absorbed energy around 100 nJ, the absorbed energy density amounts to $10^{7}$ J/cm${}^{3}$ = 10 TPa. The number of laser-affected atoms constitutes around $10^{11}$ atoms (a few picograms), making structural studies extremely difficult. Therefore, the questions arises: is it possible to increase the absorbed energy density and/or increase the amount of the laser-affected material and thus the amount of the novel phase? Preliminary studies have shown that it is very difficult to overcome the energy density of several MJ/cm${}^{3}$ (several TPa of pressure) and increase the amount of laser-affected material using a tightly-focused Gauss beam. First, the ionisation wave moving towards the laser pulse with increasing intensity increases the absorbing volume and limits the energy density [7]. Moreover, the experiments with increasing laser pulse energy demonstrated that at the energy per 150-fs pulse of 200 nJ, the cracks surrounding the focal area destroyed the regular void formation [2]. Diffraction-free Bessel beams (BB) raised the hope of achieving a higher energy density and larger amounts of the material affected. Below, we describe the recent progress made with these studies. Then, we describe some effects (and unresolved problems), the solutions of which may lead to a further increase of the absorbed energy density.

## 2. Status of the BB-Transparent Crystal Interactions

It was demonstrated recently that the BB (150 fs, 2 μJ) focused inside sapphire produced a cylindrical void of 30 μm in length and 300 nm in diameter [8]. The void volume, V=30μm $\;\times \;\pi r^{2}=2.12\times 10^{-12}$ cm${}^{-3}$, appears to be two orders of magnitude larger than that generated by the GB. The conclusions based solely on the void size measurements and on the energy and mass conservation laws without any ad hoc assumptions about the interaction process are the following [9]. The material initially filling the void was expelled and compressed into a shell by the high-pressure shock wave. The work necessary to remove material with the Young modulus *Y* from volume *V* equals at least Y×V=0.848μJ ($Y=4\times 10^{5}$ J/cm${}^{3}$, the Young modulus of sapphire). This is evidence of strong (>40%) absorption of the pulse energy. In order to generate a strong shock wave capable of expelling such an amount of material, the absorbed energy should be concentrated in the central spike with a much smaller diameter than that of the void (the absorbed energy density is still not known, theoretically nor experimentally).

The unique features of the diffraction-free Bessel beam spatial distribution of intensity in the focal area allow one to understand some of the experimental findings and indicate new problems and opportunities. The spatial distribution of intensity across the cylindrical focal volume in a transparent medium unaffected by light and observed experimentally is close to Durnin’s solution, $J_{0}^{2}\left(k_{r}r\right)$ [10]: the central spike surrounded by circular bands with the maximum of intensity on the axis approximately five-times higher than in the next band.

**Figure 1:**
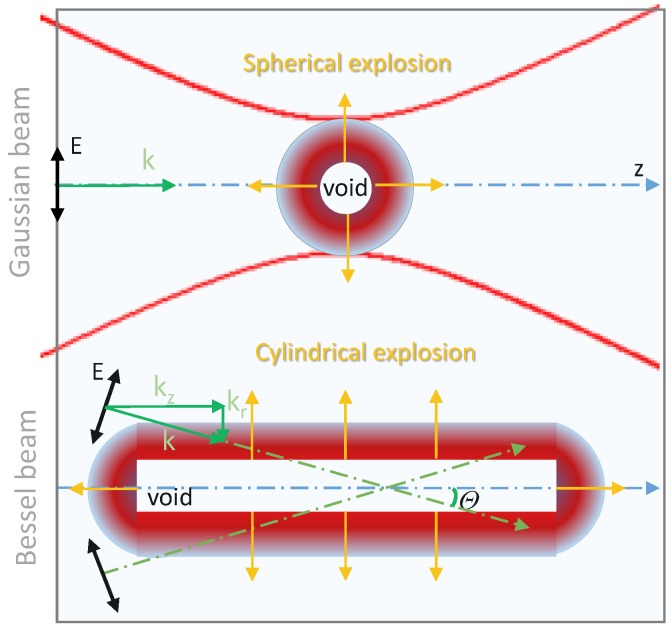
Schematic presentation of radial microexplosion-driven inside a transparent material by a focused linearly-polarised (E-field) Gaussian beam (GB) and Bessel beam (BB) with projection $E_{z}$ along the optical axis (z-axis); θ is the angle with the optical axis (wavevector $k=\sqrt{k_{r}^{2}+k_{z}^{2}}$ is shown on the upper half of the conical wave). Resonant absorption of the $E_{r}$ component (along the radial direction $k_{r}$) allows a higher energy density deposition for the cylindrical microexplosion. With the central void diameter in sapphire comparable for the GB [1] and BB [8] pulses, the volume was 180-times larger in the case of BB.

The parameters of the quasi non-diffracting BB created by any device from the incoming cw-laser pulse in air (axicon, circular slit, spatial light modulator (SLM), etc.) with the cone angle θ are the following (Figure 1): radius of the incoming beam before the BB-creating device, *R*; $z_{max}=R/\tan\left(\theta \right)$; $k_{r}=k\times \sin\left(\theta \right)$; $k_{z}=k\times \cos\left(\theta \right)$ [10]. Building the intensity distribution in the low intensity short pulse BB occurs in a similar way to that as with the cw-laser, as was demonstrated experimentally [8]. It is worth noting that in these experiments, during the pulse time, the beam propagates a distance comparable to the length of the elongated cylindrical focus ($Z_{max}$), $L_{pulse}=t_{pulse}\times c/n$ (*n* is the refractive index in a transparent crystal unaffected by a laser). For example, a 150-fs (800 nm) low intensity pulse in sapphire propagates ∼30 microns, which is close to $t_{pulse}\times c/n=30\;\mu$m [8].

In the non-absorbing media, the length of the focus (the distance where diffraction is strongly suppressed) apparently is independent of the pulse duration. On the axis of the BB (in the focal area), the time of the interaction of the electromagnetic wave with matter could be shorter than the pulse duration. Therefore, a beam of any duration allegedly propagates the same distance $Z_{max}$ allowed by the focusing device. This seemingly obvious statement should be confirmed experimentally.

**Figure 2:**
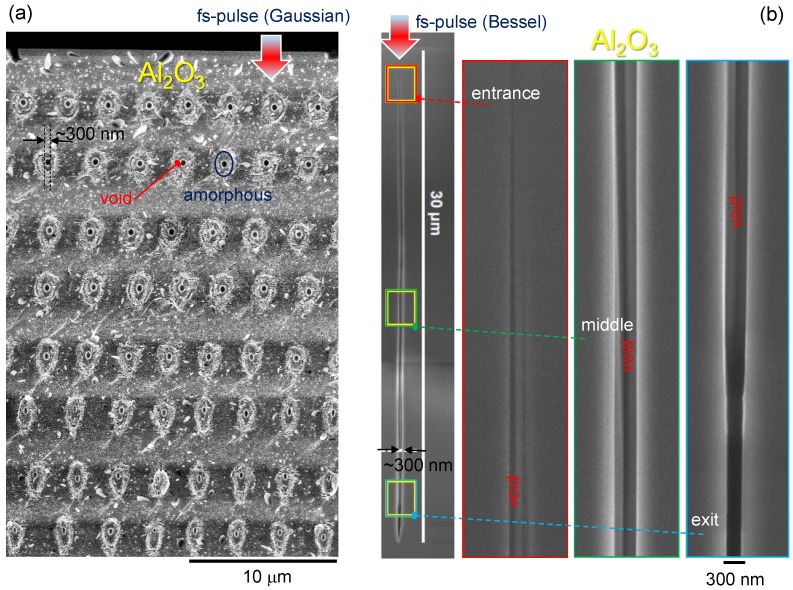
SEM side-view images of the voids made with ultra-short Gaussian [1,13] (**a**) and Bessel [8] (**b**) single pulses in sapphire. Focusing of the 800-nm/130-fs Gaussian pulses of ∼150 nJ of energy was carried out with an objective lens of numerical aperture NA=1.3 and was stacked into a vertical plane of the void-structures [13]. This plane was used to split the sapphire sample for the side-view SEM observation. The voids made at larger depth were affected by spherical aberration, which reduced the void and elongated amorphous region. The 800-nm/140-fs, 2μJ of energy, Bessel pulses were used to make cylindrical voids of a diameter of ∼300 nm revealed by focussed ion beam (FIB) milling [8].

Under the action of intense pulse, the ionization breakdown occurs early in the pulse time near the central spike where intensity is maximum. The studies of the interaction process of intense BB at an intensity above the ionization threshold are absent to the best of our knowledge. Estimates, suggestions and problems relating to the formation of the intensity distribution and interaction process based on the studies of confined microexplosion and intense short pulse interactions with dielectrics are presented below. Experimental observation of the void formation by GB and BB pulses is shown in Figure 2. The BB pulses are used to dice transparent materials [11] and to inscribe high efficiency optical gratings in silica [12].

### Control of Energy Deposition by BB Pulses

In short intense BB interaction with the initially transparent medium, the ionization threshold is reached at the axis of the focal volume where intensity attains the maximum value. It occurs early in the pulse time close to the beginning of the elongated focal region. A narrow cylindrical plasma region is created along the axis. Incident light starts absorbing in plasma. Let us take the incident field structure near the axis as the following *E* ($E_{r}$, $E_{\varphi }=0$; $E_{z}$); *H* (0; $H_{\varphi }$, 0). Then, the Poynting vector reads, $S=\frac{c}{4\pi }\left(E\times H\right)$. Therefore, the energy flows are generated inward along the radius and along the z-axis in direction of the beam propagation: $S_{r}=\frac{c}{4\pi }\left(H_{\varphi }\cdot E_{z}\right)$ and $S_{z}=\frac{c}{4\pi }\left(H_{\varphi }\cdot E_{r}\right)$ [J/(cm${}^{2}$s)].

Thus, by changing the cone angle, one can control the radial and axial energy flows. The interaction mode of intense BB with a transparent crystal dramatically changes after the ionization threshold is achieved. The surface, where the real part of the permittivity is zero, $\varepsilon_{re}=0$, separates the dielectric ($\varepsilon_{re}>0$) and plasma ($\varepsilon_{re}<0$) regions. The gradient of the permittivity is directed along the radius of a cylinder. The energy flow goes inward in the radial direction. Thus, the incident wave splits into the evanescent and reflection waves. The resonance absorption occurs in the vicinity of the zero-epsilon surface, creating a plasma wave (plasmon) propagating along the radius in the direction to the axis of the cylindrical focal region. The evanescent wave decays along the radius in the same direction. Thus, the zero-permittivity surface generates simultaneously coherent plasmons and evanescent waves coming together (focusing) to the axis of the cylindrical focal region. One may expect that coupling of evanescent waves and plasmons also contributes to the increase of the intensity and energy density near the axis in a way similar to that discovered in the studies of extraordinary optical transmission (EOT) through sub-wavelength hole arrays [14].

The ideal diffraction free beam is the monochromatic Bessel beam [10], created via superposition of plane waves, the wave vectors of which are evenly distributed over the surface of a cone. The Bessel function of the first kind zero order, $J_{0}$, is a sum of the Hankel functions of the first and second kind [15], where the inward energy flow is balanced by the outward flow.

It was suggested [16] that the quasi diffractionless BB can be presented as the result of the interference of two conical running Hankel beams, carrying equal amounts of energy towards and outwards from the beam axis, yielding no net transversal energy flux in the BB. The interference of two Hankel beams with different amplitudes creates unbalanced BB where the net radial energy flux appears. Unbalancing creates the inward radial energy flux from the conical tails of the beam. The study of stability in the frame of the non-linear Shrodinger equation (NLSE) equation revealed that the Bessel-like solutions in pure Kerr media are unstable [17].

In the interaction of intense short pulse BB with a transparent dielectric at the intensity below the ionization threshold, the BB apparently retains its balanced structure. After the plasma formation, the energy flow directed inward to the axis is created due to absorption leading to destruction of this balance. One may argue that after the ionization threshold, the Hankel function of the first kind might be considered as an appropriate approximation of the field distribution near the axis of cylindrical focus, being the exact solution of the Bessel equation describing the electric field increasing while focusing. One may conjecture that the BB becomes unstable, tending to focus onto the cylindrical axis, thus creating an energy density higher than a tightly-focused, but diffraction-limited Gaussian beam.

Let us now consider the relation between the pulse duration, absorption, focal region length and laser-affected area length. In short intense BB interaction with the initially transparent medium, the ionization threshold is reached at the axis of the focal volume where the intensity is maximum. This occurs early in the pulse time close to the beginning of the elongated focal region. The intense pulse converts the initially transparent material into strongly absorbing plasma practically at the moment of its arrival at some space point. Therefore, the plasma region gradually increases along the axis as the pulse proceeds until the end of the pulse. The last portion of light arrives after travelling through the transparent crystal a distance $t_{p}\times c/n$. The laser-affected distance then reads $L_{las}=\left(t_{p}\times c/n\right)\cos\theta$ (θ is the half-cone angle; Figure 1). One can see now the difference between the BB-affected area in a transparent medium (diffraction-free focus) and the laser-affected area in an intense short pulse laser/crystal interaction. For sufficiently short pulses, the laser-affected area might be shorter than the diffraction-free zone, $L_{las}<Z_{max}$. Thus, laser pulse duration might be another lever (along with the cone angle) to control the energy deposition volume.

Experiments demonstrated that short intense BB could affect a much larger amount of material producing solid-plasma-solid transformation (direct measurements) at allegedly a pressure of several TPa (conclusions on the basis of the analysis of the experiments) [8,9]. J.Hu [18] measured the average speed of the shock wave, $v_{sw}\approx 60$ km/s, during the cylindrical microexplosion, generated by the BB in sapphire, by the pump-probe technique. The estimate of the driving pressure based on this measurement, $P_{sw}=\varrho_{0}v_{sw}^{2}=14.4$ TPa ($\varrho_{0}$ is the initial mass density of sapphire), gives the direct experimental evidence of the extreme energy density created by the BB in the focal volume.

There are indications from theoretical studies [9] that the originally stable diffraction-free BB at high intensity in the presence of strong ionization nonlinearity may become unstable. Now, it is difficult to conclude if this may happen in a way similar to the self-focusing instability with Kerr-like non-linearity (rather, not because the paraxial approximation is invalid in this case) or similar to the instability of two unbalanced Hankel beams, which seems more relevant to the case (again, the ionization non-linearity should be accounted for).

The oblique incidence, inherent for the formation of the BB and long focus, implies the possibility of the surface wave (plasmon) formation and propagation along the zero-real-permittivity surface at the same time with the plasmon moving radially due to the resonance absorption. The plasma wave may converge to the axis, contributing to the increase in the absorbed energy density. One may conjecture if it might be relevant for some kind of Langmuir collapse.

It would be crucially important to find the electric field distribution up to the central axis in order to determine the absorbed energy density. This requires a solution of the Maxwell equations in cylindrical geometry coupled to material equations accounting for the change in the permittivity (electrons’’ number density and collision rate) in accord with the intensity in any space/time point. This is a formidable task; however, it can be clearly formulated for the numerical solution. Different approximations may also be discussed.

It was demonstrated experimentally [18] that the Bessel beam-induced microexplosion in sapphire, producing open-ended channel, proceeds as an axial-symmetric cylindrical explosion, and a mass conservation was experimentally validated [19]. Therefore, the direct theoretical modeling of the cylindrical explosion after the energy deposition of the BB beam inside a narrow on-axis cylinder also can be performed in the frame of two-temperature plasma hydrodynamics in cylindrical geometry in a way similar to as was done with the Gauss beam in spherical geometry [2].

## 3. Conclusions and Outlook

In conclusion, we should state that further progress in achieving and steering the high energy density strongly depends on the future pump-probe experiments, which will register with time/space resolution the history of the BB-generated microexplosion, processes of returning to the ambient state and new phases’ formation. It is worth showing the time and space scales for the succession of events comprising such a history that might in some approximation be extracted from the previous studies [2,4,9].

Let us suggest the BB, 2 μJ, 800 nm, 150 fs, impinges a sapphire crystal several tens of microns thick, creating a focal region of ∼30μm long at the ten microns depth from the outer surface of a sample. The stages of successive transformations are the following; the time count starts at the beginning of the pump pulse:

1. The low intensity stage before ionisation threshold lasts a few fs at the beginning of the pulse;
2. As the ionisation threshold is attained, the cylindrical plasma region is created at the axis of the focal region with a diameter less than a micron. One should note that the full length of the focal region of 30μm is reached at the end of the pulse, assuming that light propagates as in unaffected sapphire with a speed of $c/n∼2\times 10^{10}$ cm/s;
3. The cylinder diameter of the energy absorption region to the end of the pulse allegedly might be around the doubled absorption length in a dense plasma ∼60 nm;
4. The shock wave is created after the energy transfer from electrons to ions in a 7–10-ps time span;
5. The shock wave propagates during another 4–6 ps until it is converted into the acoustic wave, effectively stopped by the cold pressure of the crystal (∼Young modulus of sapphire). The void surrounded by the shell of compressed material is formed by the rarefaction wave;
6. The thermal wave of conventional heat conduction spreads into the laser-unaffected crystal, cooling the laser-affected area down to the ambient conditions during tens of nanoseconds. The material re-structuring occurs most probably during Stages 5 and 6. The whole area affected by the heat from the laser-heated region is a cylinder with a length of around 32–34 microns with a diameter of about 2–4 micrometers.

Thus, the whole area affected by the shock and heat waves from the energy deposition region is a cylinder 30 microns long and a few microns in diameter. The time span for the whole process of material transformation is around tens of nanoseconds. The recent arrival of X-ray free electron lasers (XFEL) with a pulse duration as short as 7–15 fs and a photon energy of 8–10 keV currently available at EuroXFEL at DESY in Hamburg and in SACLA XFEL at Spring-8 at Riken Institute in Japan creates new opportunities for uncovering the mechanism of the formation of the new states of matter. Up to 17-keV pulses expected in the near future at the SLAC National Acceleration Laboratory at Stanford. All these new sources or coherent ultra-short X-ray radiation will be used to uncover the processes involved in formation of such unusual material states. For such experiments, the tailored axial intensity distribution of the optical BB pulses can be prepared using diffraction optical elements [20], which can be made with a central hole for the co-axial fs-optical-pump and fs-X-ray-probe. To conclude, a light (or X-ray) probe with a sub-picosecond duration and sub-micron spatial resolution may shed light on the unusual formation of novel high-pressure phases starting from the “primeval soup” (warm dense matter) to the solid state at the ambient conditions, being preserved and confined inside a bulk of pristine crystal ready for further structural studies [7].
